# A comparative analysis of progestin-primed ovarian stimulation versus GnRH antagonists protocols pertaining to stimulation parameters and embryological outcomes in patients with endometrioma

**DOI:** 10.3389/fendo.2025.1492293

**Published:** 2025-07-08

**Authors:** Fazilet Kubra Boynukalin, Yusuf Aytaç Tohma, Meral Gultomruk, Zalihe Yarkiner, Ceren Melisa Akkaya, Sinan Ozkavukcu, Mustafa Bahceci, Gürkan Bozdağ

**Affiliations:** ^1^ Department of Infertility Clinic, Bahceci Fulya IVF Center, Istanbul, Türkiye; ^2^ Department of Obstetrics and Gynecology, Uskudar University, Istanbul, Türkiye; ^3^ Department of Infertility Clinic, Bahceci Ankara IVF Center, Ankara, Türkiye; ^4^ Department of Obstetrics and Gynecology, Atilim University, Ankara, Türkiye; ^5^ Department of Research and Development, Bahceci Fulya IVF Center, Istanbul, Türkiye; ^6^ Department of Art and Science, Cyprus International University, Nicosia, Cyprus; ^7^ Medical Faculty, Atilim University, Ankara, Türkiye; ^8^ Department of Embryology Laboratory, Bahcecei Ankara IVF Center, Ankara, Türkiye

**Keywords:** PPOS, endometroiosis, endometrioma, ICSI, GnRH antagonist

## Abstract

**Research question:**

Do embryo parameters and live birth rates differ between patients with endometrioma undergoing a freeze-all strategy using either GnRH antagonists or progestin-primed ovarian stimulation (PPOS)?

**Design:**

This retrospective cohort study was conducted at Bahceci Health Group from January 2021 to January 2023. Inclusion criteria were females aged 20–40 with confirmed endometriosis, using either GnRH antagonists or PPOS ovarian stimulation, and opting for freezing all embryos without fresh embryo transfer (ET). A total of 543 patients were analyzed, with the primary outcome being usable embryos at cleavage stage and secondary outcomes including distribution of embryo quality, clinical pregnancy, and live birth rate.

**Results:**

For the GnRH antagonist arm, the median (25th-75th percentiles) total gonadotropin dose required during stimulation was significantly higher (2725 [2100–3587.5] vs. 2400 [2050–3075] IU, p = 0.001) and duration was longer (11 [10–12] vs. 10 [9–11] days, p = 0.01), although number of mature oocytes and maturation and fertilization rates were similar in both arms. However, the linear regression analysis revealed that the number of usable day-three embryos was higher with the PPOS protocol than with the GnRH antagonist protocol (OR: 0.890, CI 95%: 0.226 – 1.554, p= 0.009). Particularly in patients that had undergone FET, the respective live birth rates were 50.0% and 54.6% in GnRH antagonist and PPOS arms, respectively, without any statistical significance (p= 0.365).

**Conclusion:**

In patients with endometrioma, the PPOS protocol over GnRH antagonists might potentially enhance the quantity of usable cleavage-stage embryos while showing no significant impact on the number of collected oocytes.

## Introduction

The European Society of Human Reproduction and Embryology (ESHRE) guidelines recommend *in vitro* fertilization (IVF) and/or intracytoplasmic sperm injection (ICSI) as effective treatments for patients with endometriosis (1). However, patients with endometriosis might have lower pregnancy rates than controls when generated from various groups of patients, especially in those with advanced disease (2–4). Although the proposed pathological mechanisms for a low chance of pregnancy are lacking, it might be due to chronic inflammation and oxidative damage and their effect on the ovarian response and quality of oocyte, which might in turn impact the potency of embryo and implantation (5).

An ongoing debate surrounds the selection of the ideal ovarian stimulation protocol for patients with endometriosis undergoing assisted reproductive technologies, as does the impact of endometriosis on IVF/ICSI outcomes. According to a systematic review evaluating 33 studies (2), women with endometrioma had a lower mean number of oocytes retrieved (SMD -0.23; 95% CI [-0.37, -0.10], 5 studies, 941 cycles, I(2) = 37%) and a higher cycle cancellation rate compared to those without the disease (OR 2.83; 95% CI [1.32, 6.06], 3 studies, 491 women, I(2) = 0%). Although the effects of prior endometrioma surgery can explain the lower ovarian response and oocyte yield by decreasing the number of available primordial follicles, independent from the diminished ovarian reserve, the effects of endometrioma/endometriosis perse on the follicles regarding steroidogenesis and its independent effect on the ovarian response remain unknown (6).

GnRH antagonist protocols result in rapid suppression of pituitary activity and offer several advantages over GnRH agonist protocols, including a shorter duration of treatment, reduced risk of ovarian hyperstimulation syndrome (OHSS), lower gonadotropin requirements, and improved patient compliance (7). Although some studies have suggested that GnRH agonist protocols may be associated with improved outcomes (8), prospective trials have not demonstrated significant differences in efficacy, and both protocols are currently considered equally effective in patients with endometriosis (9). Therefore, ovarian stimulation strategies should be individualized based on ovarian reserve markers and specific patient characteristics. Furthermore, recent evidence indicates that exposure to progestins for more than eight days may attenuate the inflammatory activity associated with endometriosis (10). This raises the hypothesis that progestin exposure during ovarian stimulation—specifically through progesterone-primed ovarian stimulation (PPOS)—may potentially enhance embryo development from oocytes retrieved during the corresponding follicular wave.

The utilization of PPOS inevitably requires the frozen embryo transfer (FET) approach due to the unsuitability of the endometrium to implantation. However, as it has been suggested that high sex steroid levels through ovarian stimulation (OS) can aggravate chronic inflammation and oxidative damage in eutopic endometrium and impair implantation, FET may be already a more rational and wise strategy than fresh embryo transfer for patients with endometriosis. Nevertheless, a recent meta-analysis including six studies revealed that FET was preferable to fresh embryo transfer with regard to a higher frequency of live births (OR, 1.53; 95% CI, 1.13-2.08; P = .007) with lower miscarriages (OR, 0.70; 95% CI, 0.50-0.97; P = .03) (11). Of note, quality of evidence was moderate, and methodological problems were evident among recruited studies.

In the current study, particularly in patients with endometrioma, we aimed to compare the effectiveness of PPOS and GnRH antagonist protocol regarding the number of available embryos on the cleavage stage as the primary outcome parameter. The distribution of embryo quality, the determinants of good-qualified embryos, and live birth rate after the first course of FET were also investigated.

## Materials and methods

### Study participants and design

This retrospective cohort study was conducted at the Bahceci Fulya IVF Center and Bahceci Ankara IVF Center from January 2021 to January 2023. It was approved by the Institutional Ethics Board (application number: 124). Its inclusion criteria were (i) females aged 20–40 years, (ii) a definitive diagnosis of endometriosis confirmed by the appearance of endometrioma cyst with ultrasonography, (iii) utilization of GnRH antagonists or PPOS ovarian stimulation protocols for pituitary suppression, and (iv) preference for freezing all available embryos without any fresh embryo transfer (ET). Patients suppressed with a GnRH agonist or treated with fresh ET were excluded. Other exclusion criteria were (i) pre-implantation genetic testing for detecting aneuploidy or monogenic disease, (ii) uterine leiomyoma destroying the cavity, (iii) congenital uterine anomalies, (iv) hydrosalpinx, or (v) history of recurrent miscarriage. Only initial cycles were included.

**Figure 1:**
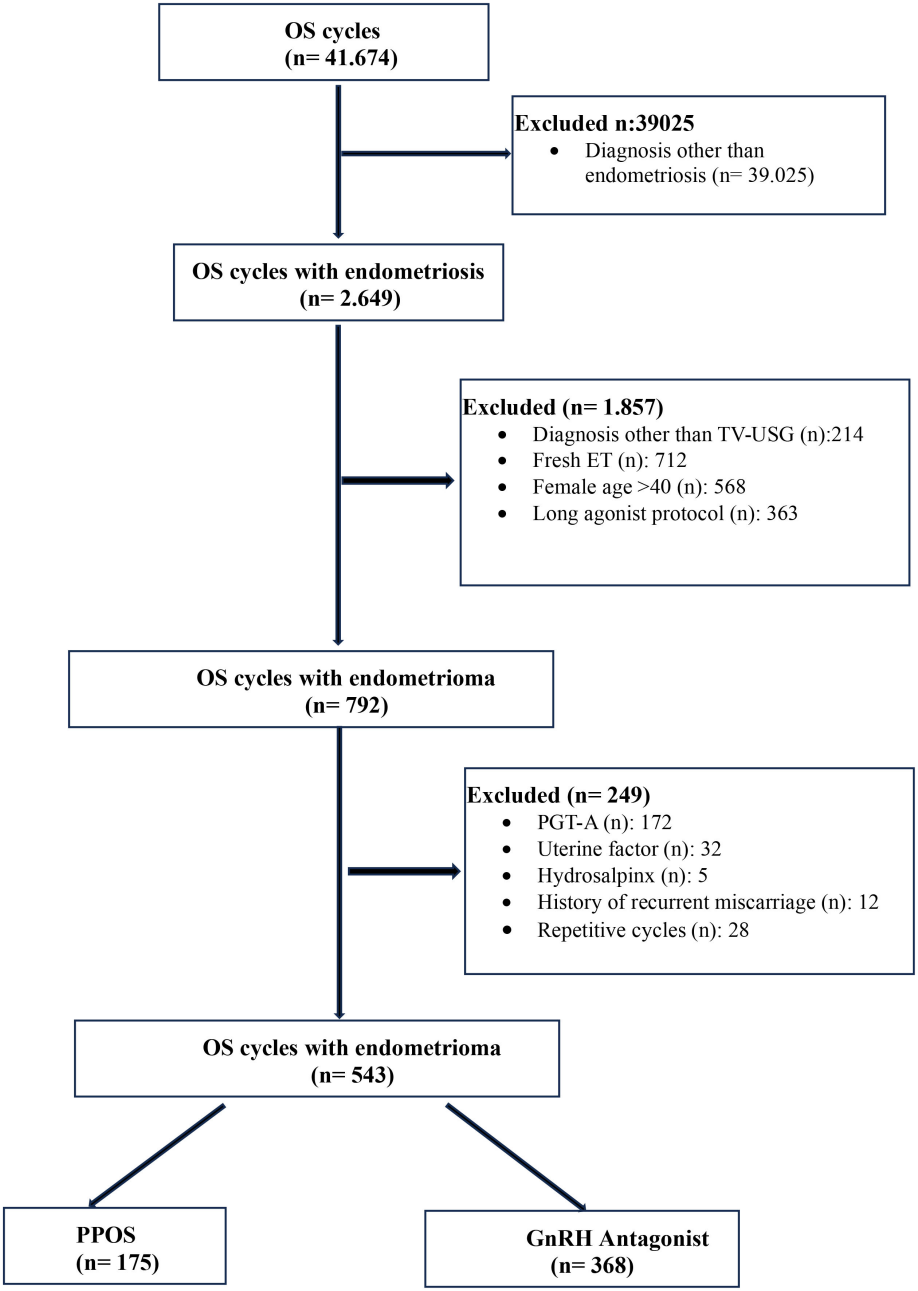
Flow-chart of the study population.

For the final analysis, a total of 543 patients were identified, of whom 368 were treated with GnRH antagonists and 175 with PPOS. Of them, 306 and 141 patients had reached the stage of FET, respectively. The flow chart representing the excluded and included patients who had been used for quantitative analysis are depicted in **Figure 1**.

### OS regimen

#### GnRH antagonist protocol

The daily gonadotrophin dosage and combination was based on the woman’s age, body mass index (BMI), and ovarian reserve. The recombinant follicle-stimulating hormone (rFSH; Gonal-F; Merck Serono, Germany) and/or purified human menopausal gonadotrophin (hMG; Merional; IBSA, Italy) was initiated on the second or third day of menstruation. The GnRH antagonist (0.25 mg cetrorelix; Cetrotide; Merck Serono,Germany) was started when the follicle diameter reached 13 mm or estradiol concentration was >200 pg/mL and was continued until the day of final oocyte maturation. Whenever the diameter of at least two follicles had reached ≥18 mm, final oocyte maturation was triggered by administering either 250 µg of recombinant human chorionic gonadotrophin or 0.2 mg of triptorelin at the specialist’s discretion.

#### PPOS protocol

Ten mg of medroxyprogesterone acetate (MPA; Tarlusal; Deva, Turkiye) was commenced daily on the second or third day of menstruation and continued until the day of final oocyte maturation. The gonadotrophin dosage was adjusted with the same methodology given in the section of GnRH antagonist. Whenever the diameter of at least two follicles had reached ≥18 mm, as measured on TV-USG, final oocyte maturation was triggered by administering either 250 µg of recombinant human chorionic gonadotrophin or 0.2 mg of triptorelin at the specialist’s discretion.

#### Embryo culture

The semen sample was collected by masturbation after two days of sexual abstinence and kept at room temperature for 30 minutes. After liquefaction and basal assessment, the sample was placed on a 45-90% double-layered density gradient medium (Isolate, FUJIFILM Irvine Scientific, Santa Ana, CA) and centrifuged at 600*g* for 10 minutes. The supernatant was discarded and the pellet was resuspended in a sperm wash medium (Sperm Washing Medium, FUJIFILM Irvine Scientific) and centrifuged at the same speed. The final pellet was resuspended in 0,5 ml sperm wash medium, observed for sperm availability, and incubated at 37°C until the ICSI procedure. After retrieval, the oocytes were incubated for two hours and denuded by enzymatic removal of granulosa cells using 80 IU/mL hyaluronidase (Hyaluronidase Solution, FUJIFILM Irvine Scientific). ICSI was the preferred fertilization method. The embryos were cultured in an equilibrated continuous single culture complete medium with human serum albumin (Continuous Single Culture Complete, FUJIFILM Irvine Scientific) in benchtop incubators (MIRI^®^ Multiroom Incubator, ESCO Medical, Egaa, Denmark) under 6% CO2 and 5% O2 throughout the culture period. The developmental stages of cleavage-state embryos were recorded on day 3 according to the Istanbul consensus (12), and blastocysts were evaluated using the Gardner and Schoolcraft scoring system (13) before cryopreservation on day 5 or 6. If there were only one or two good or moderate grade cleavage-stage embryos, they were cryopreserved on day 3. However, if more than two cleavage-stage embryos were available on day 3, they were cultured until day 5–6 and then cryopreserved. For freezing and thawing, vitrification and fast-thawing methods were used for both cleavage and blastocyst stage embryos. Selected embryos were transferred into an equilibration medium containing 7.5% DMSO and 7.5% EG for 10 minutes at room temperature. After equilibration, the embryos were moved to a final vitrification medium containing 15% DMSO, 15% EG, and 0.5M sucrose (Vit Kit-Freeze, FUJIFILM Irvine Scientific) for one minute. The embryos were then loaded onto carrier straws with minimal volume and plunged into liquid nitrogen. Thawing involved directly plunging the carrier straws from liquid nitrogen into a 37°C thawing solution containing 1M sucrose. The embryos were then kept in a dilution medium with 0.5M sucrose (Vit Kit-Thaw, FUJIFILM Irvine Scientific) for 4 minutes before being transferred into a gas-equilibrated culture medium and incubated until embryo transfer.

The available embryos on day 3 with ≥ 6 cells and fragmentation <20% were assigned as usable cleavage-stage embryos. The respective criterion for blastocyst-stage embryos were a Gardner grade of 3CC or better by day 5/6 (13). The blastocysts were categorized as follows: good (3AA, 3AB, 3BA, 4AA, 4AB, 4BA, 5AA, 5AB, and 5BA.), moderate (3BB, 3BC, 4BB, 4BC, 5BB and 5BC);, and poor (3CB, 3CC, 4CB, 4CC, 5CB, 5CC).

#### Endometrial preparation

Endometrial preparation for FET involved hormone replacement therapy with or without GnRH agonist administration. A GnRH agonist was preferred when TV-USG suggested findings of uterine adenomyosis. FET was scheduled following the first or second menstrual period after oocyte retrieval. In cases requiring pituitary downregulation with a GnRH agonist, the agent was administered during the luteal phase, and endometrial preparation commenced approximately three weeks after the onset of menses after injection. An incremental oral estrogen (Estrofem, Novo Nordisk, Turkiye) at 4 mg/day on days 1–4, 6 mg/day on days 5–8, and 8 mg/day on days 9–12 or a continuous regimen was used for estrogen priming. TV-USG was performed on the 10–13^th^ days of the cycle to measure endometrial thickness. Daily intramuscular (IM) P4 (Progestan, Koçak Farma, Turkey) was supplemented with a dose of 50–100 mg when endometrial thickness was >7mm and the serum progesterone (P4) concentration was <1.5 ng/mL. The embryo transfer was performed on the fourth or sixth day of progesterone administration if a cleavage- or blastocyst-stage embryo was planned, respectively. Oral estrogen and luteal phase support with daily IM progesterone was continued until the ninth week of pregnancy.

#### Outcome measurements

Our primary outcome parameters were the number of available embryos at the cleavage stage. Secondary outcome parameters were cancellation rate, fertilization rate, distribution of embryo quality, clinical pregnancy, miscarriage rates, and live birth rates after the first course of FET. A live birth was defined as the delivery of a liveborn baby >24 weeks and was calculated per embryo transfer.

#### Statistical analysis

Descriptive statistics were used to concisely summarize the demographic and clinical characteristics of the participants. Continuous variables were summarized using medians and 25^th^-75^th^ percentiles, including age, infertility duration, BMI, AFC, and various treatment metrics. They were compared between groups using a Mann Whitney U test. Categorical variables were compared between groups using a chi-square or Fisher’s exact tests, as applicable. Negative binomial regression was performed to evaluate the factors that affect number of usable cleavage-stage embryos. Logistic regression was used to identify predictors of the likelihood of a live birth. Statistical significance was defined at a two-tailed *p*-value of <0.05. All statistical analyses were conducted using the SPSS software (version 26).

## Results

The demographic features of patients in each group are shown in **Table 1**. Female age, duration of infertility, BMI, and AFC did not differ significantly between the GnRH antagonist and PPOS groups among patients with endometrioma. Similarly, the ratio of being in the initial or repeated IVF cycles, type of infertility, presence of adenomyosis, and concomitant diagnosis of male factor infertility were also comparable between the two groups (**Table 1**).

**Table 1:** Comparison of patient characteristics in the GnRH antagonist and PPOS groups.

Characteristics	Antagonist (*n* = 368)	PPOS (*n* = 175)	*p*-value
Age (years)	31 (29–34)	32 (29–35)	0.061
Infertility duration (years)	3 (2–5)	3 (2–5)	0.563
BMI (kg/m^2^)	23.0 (20.3–25.8)	22.7 (20.8–25.4)	0.806
AFC	9 (6–15)	9 (6–14)	0.715
Type of infertility			0.779
Primary	317/368 (86.1%)	152/175 (86.9%)	
Secondary	51/368 (13.9%)	23/175 (13.1%)	
Cycle number			
First cycle	338/368 (91.8%)	157/175 (89.7%)	0.413
Consecutive	30/368 (8.2%)	18/175 (10.3%)	
Laterality of endometriomas			
Unilateral	294 (79.9%)	137 (78.3%)	0.66
Bilateral	74 (20.1%)	38 (21.7%)	
Largest diameter of endometrioma (mm)	41 (32-52)	44 (31-49)	0.328
Adenomyosis			
Present	29/368 (7.9%)	16/175 (9.1%)	0.618
Absent	339/368 (92.1%)	159/175 (90.9%)	
Male factor infertility			
Present	100/368 (27.2%)	47/175 (26.9%)	0.938
Absent	268/368 (72.8%)	128/175 (73.1%)	

Continuous values are given as median (25^th^- 75^th^ percentiles) and were compared between groups using a Mann Whitney U test. Categorical variables were compared between groups using a chi-square test.

**Table 2** represents the differences in the OS characteristics between the GnRH antagonist and PPOS groups. The median (25^th^ – 75^th^ percentiles) OS duration was significantly longer in the GnRH antagonist group than in the PPOS group (11 [10–12] vs. 10 [9–11] days, *p* = 0.01). The total gonadotrophin dosage was significantly higher in the GnRH antagonist group than in the PPOS group (2725 [2100-3587.5] vs. 2400 [2050–3075] IU *p* = 0.001). However, the cancellation rate did not differ significantly between groups. Similarly, the endocrinological parameters of OS, the number of retrieved and mature oocytes, maturation rate, and fertilization rate did not differ significantly between groups, as shown in **Table 2**.

**Table 2:** Comparison of the cycle and embryological characteristics in the GnRH antagonist and PPOS groups.

Characteristics	Antagonist (*n* = 368)	PPOS (*n* = 175)	*p*-value
Total gonadotrophin dosage (IU)	2725 (2100-3587.5)	2400 (2050-3075)	0.001
Duration of stimulation (days)	11 (10-12)	10 (9-11)	0.01
Cancellation rate			
Yes	62/368 (16.8%)	34/175 (19.4%)	0,461
No	306/368 (83.2%)	141/175 (80.6%)	
Trigger day E2 (pg/ml)	1335 (500-2516)	1320 (430-2580)	0,719
Trigger day P4 (ng/dl)	0.58 (0.05-1.1)	0.61 (0.01-0.95)	0.334
Number of oocytes collected	10 (5-15)	9 (4-14)	0.292
Number of M2	7 (4-12)	7 (3-11)	0.313
Number of 2PN	6 (3-11)	5 (2-9)	0.489
Maturation rate	79.1 (64.6-91.1)	79.3 (66.7-90)	0.95
Fertilization rate	87.2 (70.8-100)	90.5 (72.3-100)	0.144
Cleavage-stage embryos	3 (1-6)	4 (2-7)	0.086
Blastocyst-stage embryos	2 (0-4)	2 (0-4)	0.271

Values are given as median (25^th^- 75^th^ percentiles) unless stated otherwise.

E_2_, Estradiol; P_4_, Progesterone; M_2,_Metaphase 2 oocyte; 2PN, Two pronuclei.

The numbers of usable cleavage- and blastocyst-stage embryos were similar between the groups (**Table 2**). Since the difference in the median number of usable cleavage-stage embryos was close to statistical significance, we decided to perform regression analysis to identify independent determinants of the number of usable embryos at the cleavage stage. Negative binomial regression revealed that younger age (B = –0.043, p = 0.001) and higher AFC (B = 0.033, p < 0.001) were significantly associated with increased number of usable cleavage-stage embryos (**Table 3**). Compared to the PPOS protocol, the GnRH antagonist protocol was associated with a significantly lower embryo count (B = -0.236, p = 0.023, IRR = 0.790, 95% CI [0.644, 0.968]), even after adjusting for age and AFC.

**Table 3:** Negative binomial regression model for predicting day-3 usable embryos.

Parameter	B	SE	95% CI	Wald χ²	p	Exp(B)	95% CI for Exp(B)
Intercept	2.548	0.434	[1.697, 3.399]	34.427	<.001	12.778	[5.456, 29.927]
Age (years)	-0.043	0.013	[-0.068, -0.018]	11.589	.001	0.958	[0.934, 0.982]
AFC	0.033	0.005	[0.023, 0.044]	40.069	<.001	1.034	[1.023, 1.045]
Protocol (Antagonist)	-0.236	0.104	[-0.440, -0.033]	5.183	.023	0.790	[0.644, 0.968]

The pregnancy outcomes of 447 FET cycles were examined. In the PPOS group, 141 patients had transferable frozen embryos, and all had their first FET cycle. In the GnRH antagonist group, 306 patients had transferable frozen embryos, and all had their first FET cycle. **Table 4** presents the characteristics of the FET cycles in which embryos had been generated following PPOS or GnRH antagonist OS protocol. Female age, BMI, number of transferred embryos, endometrial thickness, presence of adenomyosis, and the availability of blastocyst stage transfers were similar between the two groups. In addition, the clinical pregnancy rate, live birth rate, miscarriage rate, and multiple pregnancy rates per transfer were similar between the GnRH antagonist and PPOS groups (60.5% [185/306] vs. 59.4% [92/155], p = 0.914; 50% [153/306] vs. 54.6% [77/141], = 0.365; 17.3% [32/185] vs. 10.4% [9/86], p = 0.144; 7.6% [14/185] vs. 10.4% [9/86], p = 0.426, **Table 4**).

**Table 4:** Comparison of the first FET cycle characteristics in the GnRH antagonist and PPOS groups.

Characteristics	Antagonist (*n* = 306)	PPOS (*n* = 141)	*p*-value
Age (years)	31 (28–34)	32 (29–34)	0.081
BMI (kg/m^2^)	22.9 (20.3–25.7)	22.5 (20.8–24.7)	0.462
GnRH agonist pretreatment			
Present	25/306 (8.1%)	14/141 (9.9%)	0.540
Absent	281/306 (91.9%)	127/141 (90.1%)	
Endometrial thickness on P administration day	9.6 (7.4-10.5)	9.5 (7.7-10.2)	0.324
Number of embryos transferred (total)	403	197	
Number of embryos transferred	1 (1–1)	1 (1–1)	0.690
Adenomyosis			0.540
Present	25/306 (8.1%)	14/141 (9.9%)	
Absent	281/306 (91.9%)	127/141 (90.1%)	
Day of embryo transfer			0.803
Cleavage stage	35/306 (11.4%)	15/141 (10.6%)	
Blastocyst stage	271/306 (88.6%)	126/141 (89.4%)	
Quality of embryo			0.82
Good	197/403 (48.9%)	91/197 (46.2%)	
Moderate	170/403 (42.2%)	88/197 (44.6%)	
Poor	36/403 (8.9%)	18/197 (9.2%)	
Pregnancy outcome			
Clinical pregnancy	185/306 (60.5%)	86/141 (61.0%)	0.914
Live birth	153/306 (50.0%)	77/141 (54.6%)	0.365
Miscarriage	32/185 (17.3%)	9/86 (10.4%)	0.144
Multiple pregnancy	14/185 (7.6%)	9/86 (10.4%)	0.426

Values are given as median (25^th^- 75^th^ percentiles) unless stated otherwise.

Multivariate binary regression analysis revealed that cleavage-stage embryo transfer decreased the probability of live birth with an OR of 0.296, (95%CI: 0.147 – 0.596, *p* < 0.001) when compared with blastocyst-stage embryo transfer (**Table 5**). Other independent determinants for live birth were embryo quality (odds of good-qualified embryo vs. moderate (OR = 0.421, *p* = 0.001) or poor qualified (OR = 0.18, *p* < 0.001) and the number of embryos transferred (OR = 1.705, *p* = 0.044). However, the preference of protocol was not a significant predictor of live birth.

**Table 5:** Binary logistic regression analysis to detect independent predictors of live birth in FET cycles.

Parameters	*B*	S.E.	Wald	df	*p*-value	Exp(B)	95% CI for Exp (B)	
							Lower	Upper
Cleavage-stage embryo	−1.217	0.357	11.627	1	<0.001	0.296	0.147	0.596
Number of embryos transferred	0.534	0.265	4.043	1	0.044	1.705	1.014	2.868
Embryo quality (Good)			21.458	2	<0.001			
Embryo quality (Moderate)	−0.864	0.256	11.375	1	<0.001	0.421	0.255	0.696
Embryo quality (Poor)	−1.713	0.479	12.786	1	<0.001	0.180	0.071	0.461

## Discussion

According to our data, particularly in patients with endometrioma, PPOS might yield a higher number of usable cleavage-stage embryos than GnRH antagonist protocol. However, the live birth, clinical pregnancy, and miscarriage rates after the first FET cycle did not differ significantly when embryos had been generated with an OS of PPOS or GnRH protocols.

Although current evidence on the optimal ovarian stimulation protocol for women with endometriosis remains limited, existing studies suggest no clear superiority of one protocol over another in terms of oocyte yield, embryo quality, or live birth outcomes. This aligns with findings from the general IVF population, where both GnRH agonist and antagonist protocols—along with emerging strategies like PPOS—have demonstrated broadly comparable effectiveness. Therefore, the choice of stimulation protocol should be individualized based on patient characteristics, treatment logistics, and clinical judgment rather than presumed protocol efficacy (14).

Since the PPOS protocol was first reported in 2015 (15), it has been used with success in oocyte donation program and for the aim of fertility preservation (16, 17). Nevertheless, current evidence suggests that the PPOS protocol effectively prevents early luteinization with a similar premature ovulation rate when compared with GnRH analogue (18). The effects of the PPOS protocol on oocyte quality, maturation rate, fertilization rate, embryo quality, and the euploidy status of the embryo and live birth have also been examined in the literature. A recent meta-analysis reported that stimulation duration, total gonadotrophin dosage, and number of retrieved oocytes were similar between PPOS and GnRH analogs in different cohorts of patients (18). In addition, clinical pregnancy and live birth rates were reported to be similar between PPOS and GnRH analogs. However, a recent retrospective study suffering from some limitations reported that patients stimulated with PPOS might have inferior cumulative live birth rate when compared with GnRH antagonists (19). We must note that those findings were not confirmed with following studies ending up with comparable success parameters (20, 21).

Given the fact that endometriosis is associated with chronic inflammation that depends on estrogen, reactive oxygen species, free iron, and inflammatory cytokines released from endometrioma have been suggested to impair follicular/oocyte quality during OS (22). Of interest, progestins might have some therapeutic effects on endometriosis by modulating local estradiol biosynthesis (23) and regulating T-cell expression, which may further suppress the inflammatory process (10). However, molecular studies have shown that the anti-inflammatory effects of medroxyprogesterone acetate (MPA) become evident after eight days of exposure (10). Therefore, one may suggest that pituitary suppression with progestins during IVF/ICSI cycles might sedate the inflammation process and restore folliculogenesis, steroid synthesis, and the micro-environment around the oocyte. But still, there is a paucity of data on whether there is a clinical advantage to embryos generated after pituitary suppression with progestins during OS when compared with GnRH antagonists. To our knowledge, the current study is the first attempt with a large sample size to evaluate embryological data in patients treated with PPOS and compare them with the GnRH antagonist protocol.

There is currently limited data for the performance of PPOS in patients with endometriosis in clinical trials. Guo et al. were the first to evaluate the efficacy of using an MPA-based PPOS protocol in endometriosis cases compared with a short GnRH agonist protocol (24). Their study population included a total of 262 cycles in 244 patients with advanced stage endometriosis but a normal ovarian response. They were allocated to three groups: i) the surgery group, which included women diagnosed with advanced endometriosis by laparoscopy or laparotomy who had ovarian endometriomas that were all treated surgically before IVF and who used PPOS during OS; ii) the aspiration group, which included women who had ovarian endometriomas that were aspirated and used PPOS during OS; and iii) advanced endometriosis patients who used the short agonist protocol during OS. They reported that the numbers of mature oocytes and high-quality day-three embryos were higher in the MPA group. However, pregnancy outcomes after FET were similar between groups. They highlighted that MPA seems to be a good alternative for OS in cases that used the freeze-all strategy. Our study is distinguished from the study by Guo et al. in several ways. Firstly, we selected patients with endometrioma revealed by TV-USG to generate a more homogenous group of patients. Secondly, we compared the PPOS protocol with the GnRH antagonist protocol, which is more frequently employed in daily IVF clinic. And thirdly, our study was not specified for normal responders solely to evaluate all groups of women with endometrioma.

We found that the number of usable embryos was not significantly higher in the PPOS group than in the GnRH agonist group (4 [2–7] vs. 3 [1–6], *p* = 0.086), though it was close to statistical significance. Nevertheless, the linear regression analysis revealed that using the PPOS protocol over the GnRH antagonist protocol was positively associated with the number of usable cleavage-stage embryos, which may be due to the anti-inflammatory effect of MPA during controlled OS. In concordance with those clinical findings, a recent metabolomics analysis of follicular fluid in women with ovarian endometriosis found that those receiving PPOS had significantly lower inflammatory molecules than those receiving the ultra-long agonist protocol (25). However, it is important to interpret this finding with caution. The incidence rate ratio (IRR = 0.790) indicates a relatively small effect size, and the 95% confidence interval (0.644–0.968), although not crossing the null value, remains relatively wide, reflecting some level of statistical uncertainty. From a clinical perspective, the observed difference may not translate into meaningful improvements in patient outcomes and should be validated in larger prospective studies. We have therefore tempered our conclusions and emphasized the need for further investigation regarding the clinical relevance of this association.

Another recent retrospective study examined the reproductive outcome of patients with endometrioma undergoing IVF/ICSI-embryo transfer who had completed their first embryo transfer cycles (26). The implantation, clinical pregnancy, ongoing pregnancy, and live birth rates did not differ significantly between the PPOS and GnRH antagonist protocols within a total of 605 women with endometrioma. However, in the GnRH antagonist group, fresh embryo transfer was performed whereas in PPOS the freeze-all strategy was used. This difference may cause a bias favoring the GnRH antagonist group, since a recent meta-analysis reported better pregnancy outcome with FET in patients with endometriosis (11). In our study, both the PPOS and GnRH antagonist groups who had undergone freeze-all were recruited to compare live birth rates after the first FET cycle to exclude a potential effect of high sex steroids during OS on eutopic endometrium of patients with endometriosis. Binary logistic regression revealed that PPOS vs. GnRH antagonists was not an independent significant predictor of live birth. As the cumulative LBR was not assessed in our study, this represents a notable limitation. While the PPOS protocol was found to be an independent and statistically significant predictor of the number of usable day-3 embryos, it is important to acknowledge that embryo quantity alone does not necessarily correlate with improved reproductive outcomes. The absence of cumulative LBR data limits the clinical generalizability of our findings. Future prospective studies incorporating cumulative live birth outcomes are needed to determine whether the observed increase in embryo count under PPOS stimulation translates into actual improvements in reproductive success. We have now explicitly acknowledged this limitation and proposed it as a key direction for future research.

Inevitably, endometriosis might coexist with adenomyosis which might hamper success rates further (27). A recent meta-analysis evaluating the impact of adenomyosis on IVF/ICSI outcomes concluded that women with adenomyosis had lower LBR (OR 0.59, 95% CI 0.37-0.92, p = 0.02), clinical pregnancy rate (OR 0.66, 95% CI 0.48-0.90), and ongoing pregnancy rate (OR 0.43, 95% CI 0.21-0.88) compared to those without adenomyosis, and miscarriage rate was higher in women with adenomyosis (OR 2.11, 95% CI 1.33-3.33) (28). In our study, logistic regression analysis showed that embryo stage, embryo quality, and the number of embryos transferred were significant independent predictors of live birth in FET cycles, but adenomyosis was not found to be a significant factor. This may be related to the heterogeneity of adenomyosis, ranging from multiple lesions with diffuse myometrial hypertrophy to more severe discrete focal lesions; hence the heterogeneity in diagnosis. Therefore, the impact of adenomyosis on live births is not always the same, and the type and degree of uterine involvement may have different effects. Additionally, conducting FET cycles in both groups may have contributed to the lack of a difference in pregnancy rates, particularly among patients with adenomyosis, as this strategy had been reported to be superior to the fresh ET approach (29). But still, we aimed to discriminate patients with adenomyosis and found them to be equally distributed among the two study groups, which was not considered in the previous two studies.

Our study has several limitations. It is a retrospective study and therefore impacted by inherent drawbacks of the study design. To adjust for possible confounders, we performed regression analyses and generated a control group with similar demographic features such as female age and BMI. Secondly, we do not have any data for cumulative live birth rate, which may provide more information about the effects of PPOS on the oocyte quality and total number of cleavage-stage embryos, as mentioned above. However, while few studies have compared GnRH antagonist and PPOS protocols with fresh and freeze-all cycles, our study compared the two OS protocols in freeze-all cycles which is more accurate regarding the methodology. Nonetheless, further randomized controlled studies are warranted.

In conclusion, using the PPOS protocol for patients with endometrioma might have a positive impact on the number of cleavage-stage usable embryos when compared with the GnRH antagonist protocol. However, based on the reproductive outcomes of the first FET cycles, live birth rates are similar between the PPOS and GnRH antagonist protocols.

## Data Availability

The raw data supporting the conclusions of this article will be made available by the authors, without undue reservation.
